# Breaking paradigms in severe epistaxis: the importance of looking for the S-point^☆^

**DOI:** 10.1016/j.bjorl.2017.12.007

**Published:** 2018-01-20

**Authors:** Eduardo Macoto Kosugi, Leonardo Balsalobre, João Mangussi-Gomes, Miguel Soares Tepedino, Daniel Marcus San-da-Silva, Erika Mucciolo Cabernite, Diego Hermann, Aldo Cassol Stamm

**Affiliations:** a Universidade Federal de São Paulo (UNIFESP), Escola Paulista de Medicina, Departamento de Otorrinolaringologia e Cirurgia de Cabeça e Pescoço, Setor de Rinologia, São Paulo, SP, Brazil; b Complexo Hospitalar Edmundo Vasconcelos, Centro de Otorrinolaringologia e Fonoaudiologia, São Paulo, SP, Brazil; c Policlínica de Botafogo, Departamento de Otorrinolaringologia, Rio de Janeiro, RJ, Brazil

**Keywords:** Epistaxis, Nasal septum, Endoscopy, Natural orifice endoscopic surgery, Recurrence, Epistaxe, Septo nasal, Endoscopia, Cirurgia endoscópica por orifício natural, Recorrência

## Abstract

**Introduction:**

Since the introduction of nasal endoscopy into the field of Otorhinolaryngology, the treatment paradigm for cases of severe epistaxis has shifted toward early and precise identification of the bleeding site. Although severe epistaxis is usually considered to arise from posterior bleeding, an arterial vascular pedicle in the superior portion of the nasal septum, around the axilla projection of the middle turbinate, posterior to the septal body, frequently has been observed. That vascular pedicle was named the Stamm's S-point.

**Objective:**

The aim of this study was to describe the S-point and report cases of severe epistaxis originating from it.

**Methods:**

A retrospective case series study was conducted. Nine patients with spontaneous severe epistaxis, where the S-point was identified as the source of bleeding, were treated between March 2016 and March 2017.

**Results:**

Male predominance (77.8%) with age average of 59.3 years old were reported. Most cases presented comorbidities (88.9%) and were not taking acetylsalicylic acid (66.7%). A predominance of left sided involvement (55.6%) and anteroposterior bleeding being the principal initial presentation (77.8%) was seen. Six patients (66.7%) presented with hemoglobin levels below 10 g/dL, and four (44.4%) required blood transfusion. Cauterization of S-point was performed in all patients, with complete resolution of bleeding. No patient experienced recurrence of severe epistaxis.

**Conclusion:**

The Stamm's S-point, a novel source of spontaneous severe epistaxis, is reported, and its cauterization was effective and safe. Otolaryngologists must actively seek this site of bleeding in cases of severe epistaxis.

## Resumo

**Introdução:**

Desde a introdução da endoscopia nasal no campo de Otorrinolaringologia, o paradigma de tratamento para casos graves de epistaxe voltou-se para a identificação precoce e correta do local de sangramento. Embora a epistaxe grave seja geralmente considerada uma hemorragia posterior, um pedículo vascular arterial tem sido frequentemente observado na porção superior do septo nasal, ao redor da projeção da axila da concha média, posterior ao tubérculo septal. Esse pedículo vascular foi chamado de Stamm's S-point.

**Objetivo:**

O objetivo deste estudo foi descrever o S-point e relatar casos graves de epistaxe originando-se neste local.

**Método:**

Um estudo retrospectivo de série de casos foi conduzido. Nove pacientes com epistaxe grave espontânea, onde o S-point foi identificado como a fonte do sangramento, foram tratados no período de março de 2016 a março de 2017.

**Resultados:**

Houve predominância do sexo masculino (77,8%) com idade média de 59,3 anos. A maioria dos casos apresentava comorbidades (88,9%), mas sem uso de ácido acetilsalicílico (66,7%). Observou-se predominância do lado esquerdo (55,6%) com sangramento anteroposterior como a principal apresentação inicial (77,8%). Seis pacientes (66,7%) apresentaram níveis de hemoglobina inferiores a 10 g/dL e quatro (44,4%) necessitaram de transfusão sanguínea. Cauterização do S-point foi realizada em todos os pacientes, com resolução completa do sangramento. Nenhum paciente apresentou recorrência de epistaxe grave.

**Conclusão:**

O Stamm's S-point é relatado como uma nova região de origem de epistaxe grave espontânea, e o tratamento realizado com cauterização foi eficaz e seguro. Os otorrinolaringologistas devem buscar ativamente este local de sangramento em casos de epistaxe grave.

## Introduction

Epistaxis is one of the most common emergencies in otorhinolaryngology practice.^1^ It is extremely common in the general population; however, it is estimated that only 10% of affected individuals seek medical care, because it is usually benign and self-limited.^2^, ^3^, ^4^ Severe epistaxis, that is potentially life-threatening and requires immediate and urgent treatment,^5^ is less common, but its occurrence in specialized Otorhinolaryngology services can comprise up to 4% of the cases.^1^

Severe epistaxis is traditionally thought to arise from posterior bleeding,^5^ although the site of origen is not adequately identified in up to 50% of severe or recurrent cases.^5^, ^6^ Therefore, the usual surgical treatment of severe epistaxis involves cauterization of branches of the sphenopalatine artery, and has a high rate of success, but sometimes there is a need for an approach involving the anterior ethmoidal artery (AEA).^5^, ^7^

**Figure 1:**
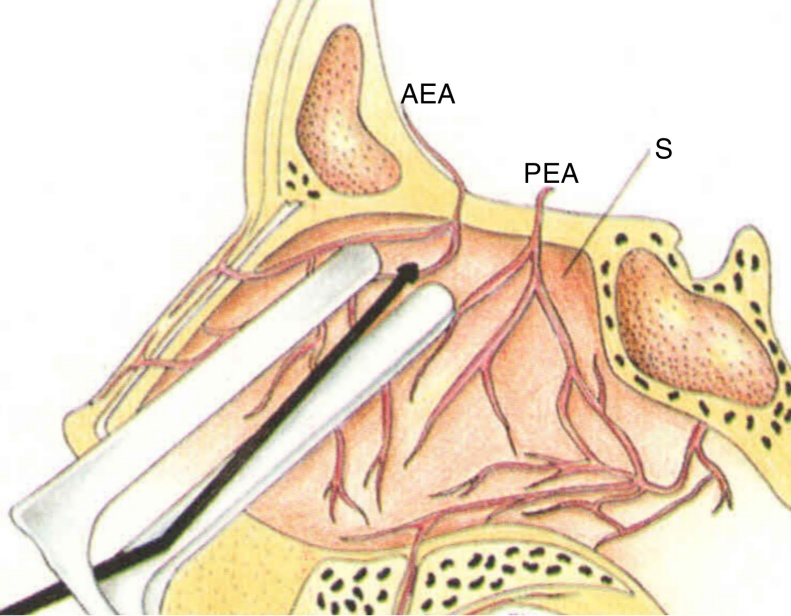
Microscopic cauterization of the branches of the anterior ethmoidal artery (AEA, anterior ethmoidal artery; PEA, posterior ethmoidal artery; S, septum).

**Figure 2:**
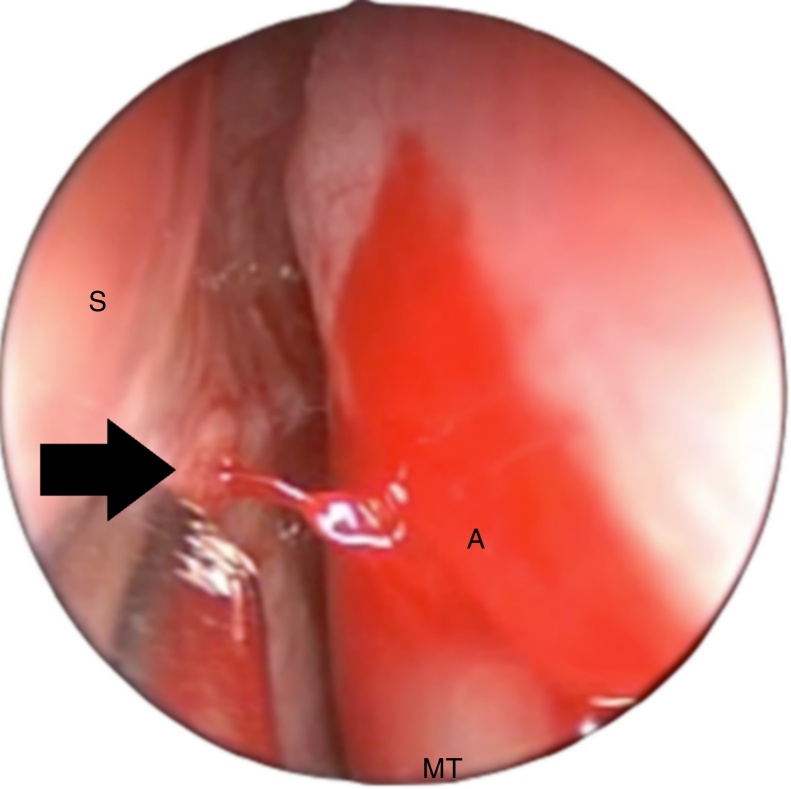
Left nasal cavity, upper endoscopic view, above the axilla of the middle turbinate (A). The S-point (black arrow) is a vascular pedicle in the upper portion of the nasal septum (S). Note that the blood pulsation can be strong enough to reach the lateral nasal wall, with a posterior flow, simulating posterior epistaxis (MT, middle turbinate).

The superior portion of the nasal septum has been considered an important site of severe epistaxis, especially the AEA branches (Fig. 1).^8^ More specifically, a bleeding point has been described by the senior author (ACS) in the upper nasal septum, around the “axilla” projection of the middle turbinate, posterior to the septal body, that presents as an arterial vascular pedicle, usually with active bleeding (anecdotal reference). This arterial vascular pedicle was named Stamm's S-point (Fig. 2).

Currently, the S-point appears to be a frequent cause of severe epistaxis. Due to its arterial nature, the S-point bleeding may be forceful enough to reach the lateral nasal wall and flow posteriorly, being mistakenly identified as posterior epistaxis (Fig. 2). Furthermore, the S-point area, much superior, located around the axilla of the middle turbinate, is not an area routinely examined by the otorhinolaryngologist during nasal endoscopic surgeries, and may be difficult to access endoscopically, since it is posterior to the septal body. Therefore, severe epistaxis originating from the S-point may not be diagnosed correctly, leading to therapeutic failures.

This report is the first to describe this specific point of bleeding in severe epistaxis. The aim of this study is to report nine patients in which the S-point was the source of severe epistaxis.

## Method

This is a series of cases of patients who experienced episodes of severe epistaxis between March 2016 and March 2017 in three different hospitals (São Paulo Hospital, Edmundo Vasconcelos Hospital, and Botafogo Polyclinic), in which the S-point was identified as the source of the bleeding.

The study was approved by the Research Ethics Committee (n. 1890166) and the participants signed the free and informed consent form.

### Inclusion criteria

Severe epistaxis with S-point origin;
Treatment with S-point cauterization;
Volunteers to participate in the study.

### Exclusion criteria

Severe epistaxis with sources other than the S-point;
Severe epistaxis of unknown origin;
Concomitant treatment with sphenopalatine artery cauterization;
Concomitant treatment with anterior ethmoidal artery cauterization.

The following data were collected: age, gender, presence of comorbidities, use of acetylsalicylic acid (ASA), epistaxis laterality, anterior and/or posterior presentation of bleeding, initial treatment performed, hemoglobin levels (Hb), presence of coagulation disorders, need for blood transfusion, surgical treatment performed and time of follow-up.

### Identification of the S-point

**Figure 3:**
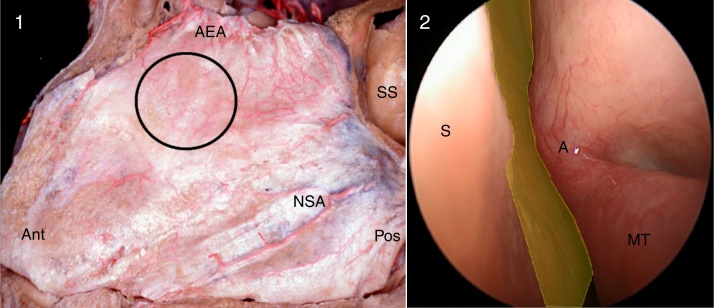
Left nasal cavity, anatomical dissection (1) and endoscopic view (2). The black circle and the yellow area show the region where the S-point (a branch of the anterior ethmoidal artery) can be found, superior to the middle meatus, in a region that is normally not assessed in nasal endoscopic surgeries (Ant, anterior; Pos, posterior; AEA, anterior ethmoidal artery; NSA, nasoseptal artery; SS, sphenoidal sinus; S, nasal septum; A, middle turbinate axilla; MT, middle turbinate).

For the correct identification of the origin of the severe epistaxis, the entire nasal cavity was carefully examined with a zero-degree endoscope, especially the superior portion of the nasal septum around the middle-turbinate axilla projection, posterior to the septal body, where the S-point is usually found (Fig. 3, Supplemental Material – video 1). For that purpose, the endoscope had to be directed to the upper region of the nasal cavity, above the middle meatus, in an area not usually accessed during traditional nasal endoscopic surgeries.

In some cases, a slight compression of the septal body using a Cottle elevator, or even septoplasty, was necessary for proper visualization of the S-point.

**Figure 4:**
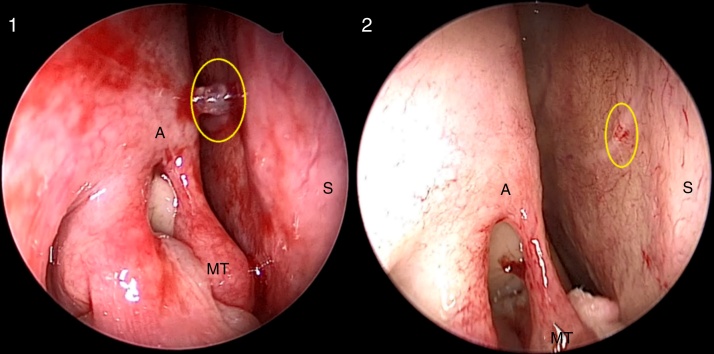
Right nasal cavity. S-point (yellow circle) before (1) and after (2) the use of cotton pledgets with topical decongestant. After vasoconstriction, the S-point vascular pedicle practically disappears (A, axilla of the middle turbinate; MT, middle turbinate; S, nasal septum).

Two other measures were necessary to identify the S-point at the initial endoscopic evaluation: blood pressure levels needed to be normal, without hypotension; and decongestant solutions could not be used until complete nasal endoscopic nasal evaluation. The use of cotton pledgets soaked in decongestant solutions could promote vasoconstriction of the arterial pedicle and temporary hemostasis, making the S-point identification impossible (Fig. 4, Supplemental Material – video 2).

## Results

Nine patients who met all the inclusion and exclusion criteria were identified: 7 men (77.8%), with a mean age of 59.3 years (median 58 years, 34–88 years). Only one patient (11.1%) had no comorbidities, and 3 (33.3%) used ASA daily due to coronary artery disease. Five patients (55.6%) bled from the left nasal cavity (LNC), with the anteroposterior presentation being the most common (7 patients, 77.8%). Anterior nasal packing was the main initial treatment (5 patients, 55.6%). Six patients (66.7%) had Hb levels lower than 10 g/dL, and 4 (44.4%) required a blood transfusion. Only one patient (11.1%) had coagulation disorders. All patients underwent cauterization of the S-point, without manipulation of the sphenopalatine and/or ethmoidal arteries. There was no recurrence of bleeding during the mean postoperative period of 10 months (median: 11 months, 2–18 months). The data are shown in Table 1.

**Table 1:** Patients’ characteristics – S-point severe epistaxis.

Patient	Age	Gender	Comorbities	ASA user	Side	Anterior bleeding	Posterior bleeding	Initial treatment	Hematological repercussion	Coagulation disorders	Blood transfusion	Follow-up period	Final treatment
1	58	M	SAH	No	Left	Yes	Yes	Anterior Packing	Hb 11.9	No	No	8 mo	S-point Cauterization
2	53	M	Hepatitis B	No	Left	Yes	Yes	Anteroposterior Packing	Hb 7.8	Yes	2 units	5 mo	S-point Cauterization
3	34	M	No	No	Right	Yes	Yes	Anterior Packing	Hb 7.2	No	No	12 mo	S-point Cauterization
4	63	M	Malnutrition, SAH	No	Right	Yes	Yes	Anterior Packing	Hb 6.2	No	2 units	18 mo	S-point Cauterization
5	71	M	SAH, MR	Yes	Right	Yes	Yes	Anterior Packing	Hb 8.0	No	2 units	18 mo	S-point Cauterization
6	58	F	SAH, Rheumatoid arthritis	No	Left	Yes	No	No	Hb 8.1	No	2 units	11 mo	S-point Caut. Septoplasty
7	71	M	SAH, MR, DM	Yes	Right	Yes	Yes	Anterior Packing	Hb 12.1	No	No	14 mo	S-point Cauterization
8	88	M	SAH, MR, DM Psoriatic arthritis	Yes	Left	Yes	No	No	Hb 11.7	No	No	2 mo	S-point Cauterization
9	38	F	SAH	No	Left	Yes	Yes	Anteroposterior Packing	Hb 9.2	No	No	2 mo	S-point Cauterization

ASA, acetylsalicylic acid; M, male; F, female; SAH, systemic arterial hypertension; MR, myocardial revascularization; DM, diabetes mellitus; Hb, hemoglobin.

### Case presentation

**Figure 5:**
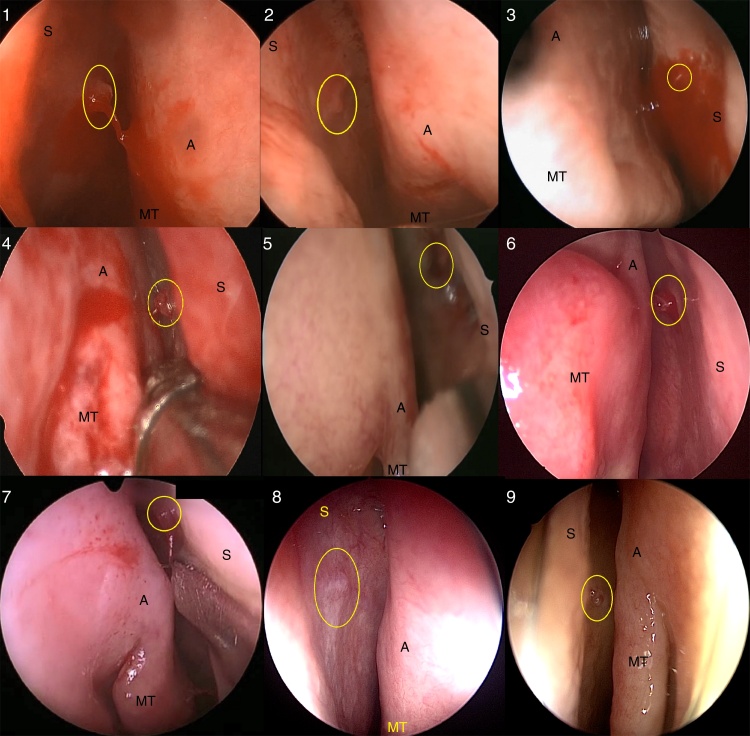
Identification of the S-point in nine cases (1–9). The S-point was identified with a yellow circle (S, nasal septum; A, axilla of the middle turbinate; MT, middle turbinate).

1st Patient: 58-year-old male, hypertensive patient, receiving atenolol and hydrochlorothiazide, with a history of severe epistaxis in LNC, was submitted to cauterization of the ipsilateral sphenopalatine and anterior ethmoidal arteries 9 months before, with surgical revision one month after the procedures due bleeding recurrence. He had no bleeding episodes for 8 months, when he had a new episode of severe epistaxis in the LNC, controlled by anterior nasal packing. A nasal endoscopy was performed under general anesthesia and active bleeding of the S-point was identified (Fig. 5(1)), which ceased after electrical cauterization. The patient has remained without bleeding episodes during the 8-month postoperative period.

2nd Patient: 53-year-old male patient with chronic hepatitis B infection was on the waiting list for liver transplantation, experienced severe epistaxis in the LNC with signs of hypovolemic shock. He was underwent hemodynamic stabilization with 3 L of lactated Ringer's solution and anteroposterior nasal packing with control of the bleeding. His hemoglobin levels decreased from 9.0 to 7.8 g/dL, with an INR of 2.12. After transfusion of two units of packed red blood cells and three units of platelets, the nasal cavity was assessed with a rigid endoscope under general anesthesia and active bleeding of the S-point was identified (Fig. 5(2)). Electrical cauterization of the S-point was performed, with control of the bleeding. The patient has remained stable during the postoperative period of 5 months.

3rd Patient: 34-year-old male patient had recurrent epistaxis in the Right Nasal Cavity (RNC) for 3 days. The bleeding was spontaneous, voluminous, but self-limited. During medical care, he had mild bleeding in the RNC, controlled with anterior nasal packing. Due to the recurrence of the condition and the presence of a serum hemoglobin level of 7.2 g/dL, endoscopic examination under general anesthesia was performed, which revealed mild active bleeding from S-point (Fig. 5(3)). The bleeding was controlled by electrical cauterization and the patient has not had any recurrences during one year of follow-up.

4th Patient: 63-year-old male patient with malnutrition and poorly-controlled systemic arterial hypertension (SAH) presented recurrent episodes of severe epistaxis in RNC in the previous 2 days. During the physical examination, he had severe nasal bleeding, controlled by bilateral anterior nasal packing. There was a decrease in hemoglobin levels on these 2 days, from 10.7 to 6.2 g/dL, requiring the transfusion of two units of packed red blood cells. The patient was taken to the OR for nasal endoscopy, which identified active bleeding from the S-point on the right (Fig. 5(4), Supplemental Material – video 3), adequately controlled by electrical cauterization. There have been no recurrences during the 18 postoperative months.

5th Patient: 71-year-old, male, well-controlled hypertensive patient, with a history of myocardial revascularization and daily use of ASA. He had already been successfully submitted to electrical cauterization of the S-point in LNC 2 months before, and now had severe epistaxis lasting one day in the RNC; it was not possible to identify the bleeding point at the emergency evaluation; the hemorrhage was controlled with anterior nasal packing. He had a hemoglobin level of 8.0 g/dL, with symptoms of acute anemia, so he received two units of packed red blood cells and was submitted to nasal endoscopy under general anesthesia. Active bleeding of the S-point in RNC was located (Fig. 5(5)) and successfully cauterized, and the patient has not had new bleeding episodes for 18 months postoperatively.

6th Patient: 58-year-old female patient, with uncontrolled hypertension and rheumatoid arthritis, using hydroxychloroquine, had had intermittent and severe, but self-limited epistaxis in LNC during the past three months. After a new episode of severe epistaxis, the patient was admitted without active bleeding. She had a previous hemoglobin level of 12.5 g/dL, which decreased to 8.1 g/dL at admission, requiring the transfusion of two units of packed red blood cells. Under general anesthesia, nasal endoscopic exploration showed the S-point in LNC with signs of recent bleeding (Fig. 5(6)). Septoplasty was performed to facilitate access and electrical cauterization of the S-point. She has had no recurrences in the 11 months of follow-up.

7th Patient: 71-year-old male patient, with well-controlled hypertension and type 2 diabetes, previous myocardial revascularization and daily use of ASA, was admitted with active severe epistaxis in RNC. The patient had longstanding recurrent epistaxis, which had worsened in the 5-day period prior to admission. The bleeding was controlled with anterior nasal packing. He had a hemoglobin level of 12.1 g/dL. Nasal endoscopy was performed under general anesthesia and active bleeding of the S-point on the right was identified (Fig. 5(7)). The bleeding was controlled with electrical cauterization of the S-point and the patient has remained stable for 14 months of follow-up.

8th Patient: 88-year-old male patient, with controlled SAH, type 2 diabetes and psoriatic arthritis, and a history of coronary angioplasty 20 years before, in addition to daily use of ASA. He had episodes of severe, intermittent and self-limited epistaxis in the LNC in the last two months, occasionally associated with hypertensive peaks. He was evaluated in the absence of a bleeding episode and nasal endoscopy was performed, which showed S-point on the left. His hemoglobin level was 11.7 g/dL, without coagulation disorders. He was admitted to undergo nasal endoscopy under general anesthesia, which confirmed the presence of the S-point (Fig. 5(8)), without active bleeding at the time of evaluation; the S-point was electrically cauterized. The patient has remained stable for 2 months of follow-up.

9th Patient: 38-year-old female, hypertensive patient, using five antihypertensive drugs. She had had frequent, intermittent, but self-limited bouts of epistaxis in LNC in the last 4 months. She was admitted with high-intensity epistaxis in LNC, and anteroposterior nasal packing was required to control the condition. Nasal endoscopic examination under general anesthesia identified the S-point with active bleeding in LNC (Fig. 5(9)). The admission hemoglobin level was 9.2 g/dL, without coagulation disorders, and there was no need for blood transfusion. Successful electrical cauterization was performed, and there has been no recurrence for 2 months postoperatively.

## Discussion

The surgical treatment of severe epistaxis has evolved from heroic measures, such as external carotid artery ligation, to procedures that increasingly value the accurate identification and control of the bleeding point. Sphenopalatine artery endoscopic cauterization is currently the most commonly performed surgical procedure for severe epistaxis, and the use of endoscopes has allowed the surgical technique refinement to increase procedural efficacy.^5^, ^9^ The popularization of nasal endoscopy has encouraged the active search for the bleeding point, previously restricted to the Kiesselbach's plexus, now with the possibility of identifying posterior bleeding points in the nasal cavity. Therefore, nasal endoscopy has resulted in an important change in paradigm regarding the treatment of severe epistaxis.^6^

There is much controversy about the location of severe bleeding cases, perhaps due to the lack of standardization in their search.^10^ Thornton et al. (2005)^11^ identified the lateral nasal wall as the main site of bleeding, with the upper nasal septum accounting for only 16.3% of the cases. Almeida et al. (2005)^6^ identified the nasal septum as the main site of severe posterior bleeding (46.6%); however, their study only identified an anterior or posterior location of bleeding, and did not report whether the bleeding was superior. Finally, Chiu and McGarry (2007)^10^ also reported that most cases of posterior epistaxis originated from the nasal septum, equally distributed between the upper and lower portions. One factor is crucial: without using the endoscope, the bleeding point might not be located in half of the cases.^5^

Although these previous studies confirmed the nasal septum as a frequent source of severe epistaxis, a specific and constant site of septal bleeding was not identified. The present study described an identifiable, stable source of bleeding in patients with severe epistaxis, located in the upper nasal septum, around the middle turbinate axilla projection, posterior to the septal body. Although it is not possible to determine prevalence rates with this study design, the S-point seems to be a frequent cause of bleeding in severe epistaxis.

Currently, the bleeding point identification and subsequent cauterization is considered an adequate, effective and safe approach in the management of severe epistaxis, being less invasive than the sphenopalatine artery cauterization.^6^ The present study characterized for the first time the S-point, a stable point of severe epistaxis, in a site not usually evaluated during nasal endoscopic examination. It has also been shown that, when identified, S-point cauterization was an effective and safe method for the treatment of severe epistaxis, even in patients with comorbidities and coagulation disorders. Therefore, the active search and identification of the S-point as the origin of bleeding can change the paradigm in the management of patients with severe epistaxis, and lead to a simpler and more effective approach.

## Conclusion

The Stamm's S-point was reported as the source of bleeding in severe epistaxis. Nine cases of S-point severe epistaxis effectively treated only by S-point electrical cauterization were reported. The dissemination of S-point knowledge as a cause of severe bleeding may increase the success of surgical treatment of epistaxis and decrease its morbidity.

## Conflicts of interest

The authors declare no conflicts of interest.

## Appendix A Supplementary data

Supplemental Material – Video 1 Left nasal cavity. Endoscopic view of the S-point origin region. The zero-degree endoscope must be directed to the upper region, around the axilla of the middle turbinate. Branches of the anterior ethmoidal artery can be seen in the upper region of the nasal septum.

Supplemental Material – Video 2 Right nasal cavity. Effect of nasal decongestants on the S-point. The use of cotton pledgets with vasoconstriction agents promotes the vascular pedicle retraction and temporary hemostasis, making it difficult to identify the S-point.

Supplemental Material – Video 3 Right nasal cavity. Active S-point bleeding. The blood pulsation may be strong enough to reach and drip down the lateral nasal wall, simulating posterior epistaxis.
